# The Utility of Machine Learning-Enhanced Developmental Cascade Models in Prevention Science

**DOI:** 10.1007/s11121-026-01897-0

**Published:** 2026-04-02

**Authors:** Vanessa Morales, Francisco Cardozo, Raymond R. Balise, Sara M. St. George, Daniel J. Feaster

**Affiliations:** Department of Public Health Sciences, University of Miami Miller School of Medicine, Miami, FL USA

**Keywords:** Developmental cascade models, Machine learning, Prevention science, Predictive modeling, Risk and protective factors, Longitudinal data analysis

## Abstract

Developmental cascade models provide a valuable framework for understanding how risk and protective factors interact over time to shape health and behavioral outcomes. Traditional statistical methods, such as logistic regression and structural equation modeling, have been instrumental in uncovering developmental pathways within prevention science. However, these methods often impose constraints on model complexity and face limitations in capturing the non-linear and interdependent nature of developmental processes. Machine learning (ML) offers complementary advantages, such as the ability to incorporate high-dimensional data, detect complex interactions, and enhance predictive accuracy. These capabilities can improve identification of at-risk individuals, support the timing of interventions across developmental stages, and refine theory-driven models. By integrating ML with developmental cascade models, researchers can more effectively identify when and how which risk accumulates and protective factors exert influence, thereby improving the tailoring and efficiency of prevention strategies. This conceptual paper outlines how ML can extend traditional analytic approaches in developmental cascade research, discusses key practical considerations for researchers including data requirements, software selection, and model validation, and highlights its potential to advance prevention science across the life course.

## Introduction

### Developmental Cascade Models for Prevention Science Research

Developmental cascade models hypothesize that early life risk factors interact and accumulate across successive stages of development which produce a chain of influences that shape long-term health and behavioral outcomes (Otten et al., 2019). Evidence from existing cascade research highlights four key principles regarding how risk and protective factors operate, as well as how interventions can alter developmental pathways (Smith et al., 2018). These key principles include (1) changes at any single point in the cascade can have broad downstream consequences; (2) patterns of behavior and environmental influences exhibit continuity from early childhood through adolescence; (3) individual risk or protective factors generally exert small effects, but their cumulative impact can be substantial, suggesting that modest increases in protective factors may yield significant long-term benefits; and (4) early developmental experiences are particularly important because they set in motion processes that perpetuate and compound over time (Smith et al., 2018). Together, these key principles align with the core principles of prevention science, as identified by the Society for Prevention Research, namely, the importance of developmental timing, ecological influences, and the transactional nature of risk and protective factors (Gottfredson et al., 2015).

**Fig. 1:**
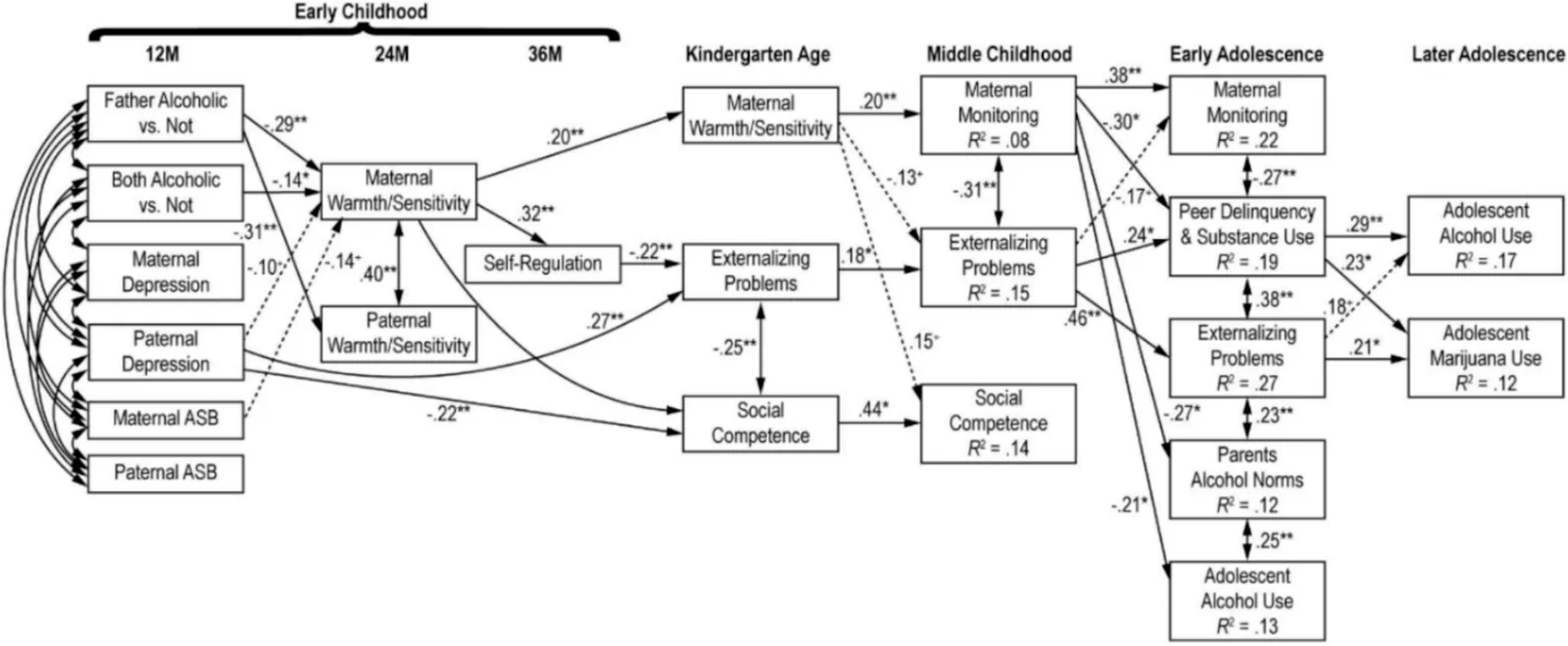
(Eiden et al., 2016)

Complex prevention problems are inherently multilevel and dynamic, warranting transdisciplinary approaches. Developmental science remains underutilized in many such efforts, despite its focus on timing, context, and transactional processes (Harrist et al., 2012). Previous research has applied developmental cascade models to predict outcomes such as externalizing problems in fourth grade, substance use during adolescence, and autonomy in adulthood (Lier & Koot, 2010; Oudekerk et al., 2015; Wolchik et al., 2016). For example, when applied to substance use prevention, Wolchik et al. (2016) demonstrated how a parenting-focused preventive intervention in childhood led to fewer mental health problems, lower substance use, and higher competencies in adolescence, which in turn predicted lower internalizing and externalizing problems, reduced binge drinking, and higher academic competence in emerging adulthood (Wolchik et al., 2016). Similarly, Eiden et al. (2016) presented a developmental cascade model illustrating how early experiences, such as prenatal warmth and child self-regulation, shape long-term health and behavioral trajectories, influencing adolescent substance use and delinquency (Eiden et al., 2016). Figure 1 reprinted from Eiden et al. (2016) depicts this model as an example of a developmental cascade process. These applications have empowered prevention scientists to gain a more nuanced understanding of the interdependent relationships among risk and protective factors and identify developmental windows for intervention. A developmentally informed perspective is essential for designing and implementing prevention strategies that are well-timed, resource-efficient, and maximally effective for preventing the onset of adverse health outcomes across the life course.

Translating these theoretical principles into empirical research requires longitudinal, multi-wave designs that can capture how early experiences across multiple socio-ecological domains (e.g., individual, peer, family, school, and community) shape later outcomes through cascading effects (Masten & Cicchetti, 2010; Smith et al., 2018). For example, one study monitored children from ages 2 to 14 to examine how early-life stress predicts adolescent substance use (Otten et al., 2019), while another followed families for 15 years after a childhood intervention to assess outcomes in emerging adulthood (Wolchik et al., 2016). Additionally, cascade studies often focus on at-risk or community samples, where higher variability in outcome measures may be present, which increases the likelihood of observing developmental cascades (Conduct Problems Prevention Research Group, 2011; Kenneth A Dodge et al., 2008; Otten et al., 2019). These methodological characteristics provide a robust framework for testing cascading processes and identifying mechanisms of developmental change over time. Given the complex nature of these cascading mechanisms, it is important to consider how developmental cascades are analyzed statistically and what innovations can be employed to capture these dynamic processes.

### Traditional Statistical Approaches to Evaluate Developmental Cascade Models

Developmental cascade models differ from traditional statistical models in that they do not rely on predefined equations to specify fixed mathematical relationships between variables. In conventional approaches such as linear regression, the relationship between variables is typically modeled as static and linear, defined by an equation such as *y* = *β*₀ + *β*₁*x* + *ϵ*, where the coefficients (*β*) represent fixed effects between predictors and outcomes (Schober & Vetter, 2021). While their empirical evaluation still requires quantifying relationships through statistical analyses, developmental cascade models are inherently process-oriented rather than equation-bound, allowing researchers to explore developmental interdependencies that unfold across multiple contexts and time points (Smith et al., 2018).

Traditional modeling approaches, such as logistic regression, multinomial regression, and Poisson regression, have long been foundational to prevention science research (Wiedermann et al., 2023). A recent review of articles published in *Prevention Science* in 2019 and 2020 found that most studies exploring categorical outcomes relied on logistic regression or multinomial logistic regression (77% and 80%, respectively), with a smaller proportion using Poisson regression (32%) or linear or structural equation modeling (37%) (Wiedermann et al., 2023). These approaches have advanced our understanding of health and behavioral outcomes; however, they rely on several assumptions, including linearity, additivity, independence of errors, and homogeneity of effects across populations (Babyak, 2004; Osborne, 2015). Such assumptions are often violated in developmental data, which are longitudinal, interdependent, and subject to time-varying influences (Gibbons et al., 2010). For example, in the case of pediatric obesity, logistic regression can identify baseline risk factors at a single time point but it is less equipped to capture how those factors interact dynamically as children grow and encounter shifting social and environmental influences (Bzdok et al., 2018; Smith et al., 2018). Consequently, traditional methods can yield static, population-averaged predictions that obscure meaningful heterogeneity in developmental pathways.

To overcome these limitations, prevention scientists have adopted flexible longitudinal modeling techniques, such as latent change score models, cross-lagged panel designs, and structural equation modeling. Latent change score models focus on differences between successive measurement points, allowing researchers to model cumulative effects of early risks on later outcomes (Klopack & Wickrama, 2020). Cross-lagged panel designs permit testing bidirectional relationships, revealing whether constructs predict each other over time (Baribeau et al., 2022). Within SEM frameworks, path analyses are often used to examine cascading mediation and moderation processes, assessing how intervening variables transmit or modify effects across developmental periods (Dishion et al., 2010; Kenneth A. Dodge et al., 2008; Smith et al., 2018). Latent growth and parallel process models further enable examination of co-developing constructs (Kwok et al., 2024; Lee et al., 2018). Even these advanced frameworks still require a priori model specification and are limited in their ability to detect higher-order interactions or nonlinearities across multiple developmental domains without guidance (Bzdok et al., 2018; Hamaker et al., 2015).

Although each of these approaches supports empirical testing of developmental cascades, they vary in their flexibility and scalability and may yield population-averaged inferences that can mask heterogeneity in developmental pathways (Molenaar, 2004). These limitations underscore the need for analytic approaches capable of extending these efforts. Specifically, ML can complement traditional approaches in two key ways: (1) exploratory feature selection to identify variable interactions both in the classical statistical sense—where the effect of one variable depends on the level of another—and in a broader, more nuanced way that captures complex, heterogeneous relationships across subpopulations, nonlinear effects, or context-dependent patterns that are most predictive but not apparent in pre-specified models (Pudjihartono et al., 2022) and (2) classification and prediction, to train algorithms to estimate future outcomes or identify at-risk individuals, providing a quantitative check on the robustness of developmental theories (Black et al., 2023).

### Advantages of Machine Learning

Extending this foundation, ML approaches address limitations of traditional statistical modeling. Fundamentally, ML methods serve two complementary functions: feature selection and prediction/classification. Feature selection algorithms can be used to identify which risk and protective factors most strongly predict developmental outcomes, while predictive models can generate individualized forecasts of risk, resilience, or treatment response. This dual capacity allows researchers to evaluate developmental theories by uncovering influential predictors and evaluating how well those predictors forecast outcomes across time.

Beyond these complementary functions, ML offers a range of additional advantages that further extend the analytical power of developmental cascade models. In this section, we highlight how ML methods address challenges faced by traditional analytic approaches such as handling complex, high-dimensional developmental data and overcoming methodological limitations that restrict the detection of nonlinear and interactive effects.

Integrating ML with traditional statistical frameworks thus represents an opportunity to enhance developmental cascade research. By combining the interpretive strengths of traditional models with the predictive and pattern-detection power of ML, researchers can refine existing theoretical frameworks, uncover novel pathways, and better capture the cumulative and dynamic nature of human development.

#### Leveraging High-Dimensional Data and Multi-domain Data

Cascade frameworks recognize that multiple domains, from biological factors to family dynamics and peer influences and school environments jointly shape developmental outcomes. As a result, cascade models tend to be more complex, with many interrelated predictors. Traditional SEMs (Byrne, 2016), among the most common approaches used in cascade research, often restrict the analysis to a limited subset of predictors due to concerns about model identifiability and over-parameterization. In contrast, ML algorithms can incorporate dozens or even hundreds of predictors with few constraints (Ribeiro et al., 2016; Tibshirani, 2011). For instance, whereas an SEM might use composite latent factors for family functioning or neighborhood risk, an ML model can incorporate individual-level variables (e.g., self-regulation), family indicators (e.g., parental warmth), and contextual features (e.g., school safety indices) simultaneously in a single predictive framework, without imposing a predefined hierarchical or latent structure, to explore how multilevel features jointly shape the broader “risk ecosystem” influencing youth outcomes. Regularization methods such as LASSO penalize model complexity, allowing high-dimensional estimation even when the number of predictors exceeds the sample size (Tibshirani, 2011). Similarly, Gaussian process regression and related kernel-based approaches provide flexible, data-efficient modeling options for nonlinear developmental trajectories (Rasmussen & Williams, 2005).

#### Capturing Non-linear Relationships and Interactive Developmental Processes

Developmental processes are rarely linear. ML methods such as decision trees (Quinlan, 1986), random forests (Breiman, 2001), neural networks (Goodfellow et al., 2016), and more modern methods excel at identifying threshold, curvilinear, or synergistic effects that traditional regression models must pre-specify (Hastie et al., 2009; James et al., 2021, 2023). For example, analyses of medication adherence have shown that ML approaches can detect complex interactions among sociodemographic, psychological, and behavioral factors (Masiero et al., 2023; Zakeri et al., 2022). Unlike traditional cascade modeling approaches, which require the a priori specification of interaction terms, ML can reveal these moderation effects directly from the data. This data-driven decision process enriches the conceptualization of risk pathways and opens new avenues of research for disentangling effects, thereby offering a broader view of how various risk factors interact.

#### Enhancing Predictive Power and Prevention Precision

The purpose of prevention is not only to explain outcomes but also to accurately predict who is at greatest risk so that interventions can be effectively tailored. ML algorithms can be explicitly optimized for predictive accuracy through techniques such as cross-validation (Arlot & Celisse, 2010), which iteratively partitions data into analysis and assessment sets to evaluate performance under different parameter settings (Kuhn & Silge, n.d.). These methods enable the generation of individualized risk profiles or prototypes showing how specific constellations of risk and protective factors influence predicted outcomes (see Biecek and Burzykowski (2021) for an approachable introduction) (Biecek & Burzykowski, 2021). Recent developments in uncertainty quantification (e.g., conformal prediction) further allow researchers to attach confidence bounds to predictions, improving their utility for clinical or practical decision-making (Vovk et al., 2005). Such personalized predictions contrast with the group-level parameter estimates typical of regression or SEM, highlighting ML’s ability to tailor interventions to individual developmental trajectories.

#### Making Models Transparent and Interpretable

A common critique of ML is its “black-box” nature. However, advances in explainable AI (XAI) provide transparent methods for interpreting complex models. Tools such as Breakdown Plots, SHAP (Shapley Additive exPlanations), LIME (Local Interpretable Model-Agnostic Explanations), or ceteris-paribus (CP) methods quantify how each predictor contributes to a given prediction, enabling researchers to visualize which variables most strongly drive outcomes for different subgroups (Biecek & Burzykowski, 2021; Lundberg & Lee, 2017; Molnar, 2022; Ribeiro et al., 2016). For example, SHAP plots can reveal that peer deviance exerts a stronger influence on substance-use risk for early-maturing boys than for girls, providing theoretically meaningful, subgroup-specific insights. In this way, XAI techniques bridge predictive analytics and theory development by showing why an ML model makes a particular prediction, information that is rarely available from traditional methods. Other XAI methods like ceteris-paribus profiles and ceteris-paribus oscillations afford the ability to explore how variables differentially impact “prototypical” people. For example, these methods may illustrate how family income differentially impacts the predicted probability of obesity development or future substance use for youth with distinct demographic profiles (e.g., a preteen White rural boy versus a teenage Black Hispanic urban girl).

#### Advancing Theory Through Data-Driven Discovery

Rather than replacing theory, ML complements it by uncovering patterns that refine and extend theoretical models. In developmental cascade research, discrepancies between predicted and observed outcomes are not failures but opportunities to identify missing mediators, moderators, or nonlinear mechanisms. This iterative theory-refinement cycle uses data-driven discovery to adjust conceptual models, which in turn improves our understanding of the complex interplay among developmental factors. In this way, predictive accuracy not only validates existing models but also illuminates paths for future theoretical refinement. This method advances prevention science by linking empirically derived interactions back to developmental theory.

#### Limitations and Challenges of ML in Prevention

Despite these advantages, ML approaches present notable challenges. They require large, high-quality datasets for optimal performance (Beam & Kohane, 2018; Bzdok et al., 2018; Ellis et al., 2022), which can be difficult to obtain in longitudinal or interventional research. For example, randomized controlled trials often have relatively small sample sizes, requiring the harmonization of multiple datasets to afford the opportunity to apply ML methods. ML models may also struggle with class imbalance, which is a common issue when outcomes are rare, and may overfit if data are limited or poorly curated (Saito & Rehmsmeier, 2015; Van Den Goorbergh et al., 2022). While algorithms exist to address these concerns, analysts must spend considerable time evaluating the impact of preprocessing steps, such as downsampling the majority class to match the rare class (reducing the number of cases in the common class) or upsampling the rare class (increasing the number of cases in the uncommon class) (*GitHub—Tidymodels/Themis: Extra Recipes Steps for Dealing with Unbalanced Data*, n.d.). Although these techniques can potentially help improve performance for rare cases, they should be applied thoughtfully. When used appropriately, they can mitigate issues arising from class imbalance, which would otherwise produce biased models that are uninformative for prevention. At the same time, these methods may introduce challenges in certain contexts, including potential biases for interference (Van Den Goorbergh et al., 2022). Furthermore, complex algorithms demand substantial computational resources and careful parameter tuning. Addressing these challenges requires thoughtful model selection, robust validation, and transparency regarding model performance and generalizability, all of which demand considerable time and effort.

### Practical Considerations for Applying Machine Learning to Developmental Cascade Research

We present recommendations for researchers aiming to apply ML to developmental cascade research. To effectively evaluate developmental cascade models, data must be longitudinal, capture a diverse range of mediating and moderating variables across key developmental stages, and have sufficiently large sample sizes to ensure robust statistical power (Smith et al., 2018). Examples of datasets that meet these requirements can be found in large-scale longitudinal studies like the Adolescent Brain Cognitive Development study (Saragosa-Harris et al., 2022), the Fragile Families and Child Wellbeing Study (Geller et al., 2018), the Environmental influences on Child Health Outcomes study (LeWinn et al., 2022), and the National Longitudinal Study of Adolescent to Adult Health (Harris et al., 2019), all of which are publicly available upon request. In addition to selecting the right data source or designing studies that meet these criteria, researchers must also select the right software and tools and adopt workflows that facilitate data preprocessing, model building, and validation. Beyond data sufficiency, researchers should ensure that the selection of predictors and outcomes remains guided by developmental theory. Theory-informed feature selection helps focus ML models on constructs that have conceptual meaning within prevention science (e.g., family processes, peer influences, self-regulation), improving both interpretability and theoretical alignment.

The choice of software for ML can significantly impact both the efficiency and reproducibility of analyses. While commercial software venders offer expensive tools for conducting ML, most ML researchers rely on the programming languages R and/or Python to solve real-world problems. In particular, R’s Tidymodels framework (Kuhn & Frick, 2022; Kuhn & Silge, n.d.) provides a cohesive and modular approach to building ML models, making it ideal for handling the complex data structures typical of longitudinal studies spanning multiple developmental periods. That said, R is a relatively niche tool, favored by academic statisticians developing new methods and by statistical programmers specializing in data visualization. Python, in contrast, consistently ranks as number one or two in the major indices of programming language importance (*The Top Programming Languages 2025—IEEE Spectrum*, n.d.; “TIOBE Index,” n.d.). Python’s popularity as a general-purpose programming language, along with its ML tools such as TensorFlow, Keras, and Scikit-Learn (*Keras*, n.d.; *Scikit-Learn*, n.d.; *TensorFlow,* n.d.), makes it widely used for ML deployment (Pedregosa et al., 2012). Python’s ML libraries and natural language processing capabilities are mirrored by R’s tools, including Tidymodels, mlr3, torch, tidytext, textrecipes, and textfeatures (Hvitfeldt & Silge, 2021; Lang et al., 2019). Thus, both languages are popular options.

In prevention science, R remains ideal for integrating ML with traditional statistical workflows driven by statisticians, whereas Python is an excellent choice in environments with analysts trained in computer science. Ultimately, the choice of programming environment should reflect the research question, team expertise, and available computational resources. Below, we enumerate the steps and describe the many interrelated packages typically used when doing ML work in R. Python users generally build similar workflows using tools within the Scikit-Learn package.

#### Model Specification

The first step for any ML project in tidymodels is to define the model specification (Kuhn & Frick, 2022). In this phase, you declare the type of model you intend to use (tree-based model, a regularized linear model, a neural network, etc.) and set its initial parameters. Initially, the hyperparameters (values that govern model behavior and are defined prior to training) are typically set to default values, but are later optimized to search for hyperparameter values that maximize model performance (Kuhn & Frick, 2022).

#### Data Preparation with Recipes

Once the model is specified, a recipe (Kuhn & Frick, 2022) can be used to create a preprocessing blueprint. This stage is critical because it transforms raw data into a format suitable for modeling. In a recipe, you can include a wide array of steps such as imputing missing values, normalizing or standardizing continuous variables, encoding categorical variables, and even creating interaction terms or composite features. The flexibility of recipes allows you to tailor the data preparation process to accommodate the needs of different models, ensuring that all predictors are appropriately handled.

#### Data Splitting into Training and Testing Sets

Before model estimation, it is essential to divide the dataset into training and testing datasets. The training set is used to build and fine-tune models, while the test set is reserved for a single final evaluation to assess the utility of the “best model.” Because the testing data is not used during model training to build the model, it provides an unbiased estimate of how well the model is likely to perform on new, unseen data.

#### Fine-Tuning and Hyperparameter Selection

After specifying the model and preparing the training and test data, the next step is to optimize a model’s performance through tuning. This involves taking portions of the training data (sometimes called an analysis subset of the training data) and building a model with that data and then checking its performance on a different portion (sometimes called the assessment subset of the training data). Tuning involves trying different model-specific parameters, called hyperparameters, and selecting the values that give the best predictive performance. For example, a *K*-Nearest Neighbors algorithm tunes the number of similar individuals (*k*) used to make a prediction. It may first evaluate how well the model predicts a new individual using the three most similar people (*k* = 3) and then compare performance when using more but less similar neighbors (*k* = 10). All this work requires many subsets of the training data. Common methods for building analysis and assessment samples of the training data include cross-validation, bootstrapping, related techniques (James et al., 2021).

Beyond modelling accuracy and the methods that are traditionally emphasized in the social or medical sciences (i.e., sensitivity, specificity, or RMSE), there are many additional performance metrics that can be used to evaluate and optimize ML models (Opitz, 2024; Sokolova & Lapalme, 2009). For example, when class sizes are imbalanced, the Matthews Correlation Coefficient provides a more reliable summary of true and false positives and negatives than overall accuracy. Log Loss imposes stronger penalties on weak predictors, while the Precision–Recall AUC prioritizes performance on the positive class. In addition, Cohen’s Kappa highlights performance in situations where chance agreement is high, making it particularly valuable when categories are highly imbalanced (Balise et al., in press). These metrics help researchers assess model performance in settings where traditional metrics may be misleading, such as when outcomes are rare or classes are imbalanced. The performance metrics selected should align with your research objectives and the nature of your outcome variable and should consider imbalanced data (i.e., rare outcomes); common in prevention studies where adverse outcomes are rare, metrics like those listed above should be considered and evaluated to tune models (Kuhn & Silge, n.d.).

#### Model Estimation and Testing

Typically, many ML algorithms are tried, such as random forests, LASSO models, and a Neural Network. Using the analysis and assessment subsets of the training data, each of these models is optimized according to an evaluation metric, such as accuracy or ROC AUC. The model with the best assessment metric (for example, highest accuracy) is declared the “winner.” The next step is to estimate this model’s behavior on test data. This estimation provides interpretable outputs, such as model coefficients or variable importance scores, which can offer insights into the relationships among variables. It is crucial that the test data is used only once, exclusively for final evaluation, to avoid overly optimistic estimates of future performance. After performance is evaluated, interpretation is critical. Explainable AI (XAI) techniques such as Breakdown Plots, SHAP, LIME, or CP methods can be applied to visualize how predictors influence model outputs, thereby connecting data-driven findings back to developmental theory. Whenever possible, models should also be validated on an independent sample or through temporal or site-based cross-validation to assess generalizability beyond the training dataset.

#### Workflow Integration

One of the most attractive features of the state of the art ML analysis tools like R’s Tidymodels is its ability to integrate the model specification, data recipe, and tuning process into a workflow (Vaughan et al., n.d.). This integration allows you to experiment with different models, adjust preprocessing steps, and iterate on hyperparameter settings in a reproducible manner. The workflow abstraction streamlines the entire process, from data cleaning and preprocessing to model building and validation, making it easier to compare various ML algorithms and select the best-performing model. This workflow makes it possible to do tasks like building and assessing the quality of predictions with a logistic regression and then seamlessly swapping out the logistic engine and then assessing a neural network, decision tree, or other modeling machines, thus, allowing researchers to find the best possible predictions.

Integrating ML workflows within developmental cascade research enables prevention scientists to move fluidly between theory, data preparation, and prediction. By embedding these steps in reproducible pipelines, researchers can iteratively test how early risk factors, mediators, and moderators operate across time and context. This process not only enhances prediction accuracy but also supports the continuous refinement of developmental theories through empirically derived insights.

### Longitudinal and Cascade-Specific Considerations

While ML frameworks have historically been built for cross-sectional prediction and therefore do not explicitly handle the correlation structure within longitudinal data, this is an area of active work. In the last five years, code to enable population-averaged effects (GEE) and subject-specific effects (mixed-effects models) in ML workflows has started to appear (Kuhn & Frick, 2022). Similarly, Monte Carlo-style cross validation methods that account for grouping variables were recently introduced and will ultimately enable estimation with repeated measures (Frick et al., 2022). While multistate models to track individuals through time are starting to appear, these methods are still in their infancy (Rickert, 2023). Until they mature and evolve, researchers using ML methods will need to focus on cross-sectional prediction of specific times or ages in the future.

In addition, most existing cross-sectional ML approaches can be utilized with careful feature preparation. For example, estimates of levels and changes between different periods can be used to predict subsequent outcomes (Liu et al., 2023). These features can be constructed over multiple periods to predict a final outcome, or as a series of sequential ML models to explore specific sub-periods of the cascade in cascade-based research designs. Such an approach allows investigators to model transitions between stages of the cascade while maintaining compatibility with standard ML workflows.

Furthermore, resampling strategies can be used in longitudinal ML applications to structure model training and evaluation in ways that respect temporal dependence. For example, blocked, clustered, or rolling resampling procedures can be used to ensure that observations from the same unit or time period are not split across training and validation sets, thereby preserving temporal ordering and within-unit dependence (Kuhn & Silge, n.d.).

Finally, different classes of ML models may be better suited to different research questions. Cross-sectional modeling frameworks may be appropriate when the objective is prediction at a specific time point or age within the cascade. In contrast, time-series or sequential modeling approaches may be better aligned with questions focused on temporal dynamics, stage transitions, or progression through cascade states. Explicit consideration of the research objective, whether prediction at a fixed future time, modeling change between defined periods, or tracking movement across cascade stages, can help guide selection of the most appropriate class of ML models.

## Conclusion

This conceptual paper explored the integration of ML with developmental cascade models to advance prevention science research. By combining the predictive flexibility of ML with a developmentally grounded framework, this approach enables researchers to empirically test complex, longitudinal relationships that are often hypothesized but rarely examined in full. For example, ML could be applied to evaluate frameworks such as the Conceptual Developmental Cascade Models for Pediatric Obesity proposed by Smith et al. (2018), or the Six C’s model for childhood obesity (Harrison et al., 2011), both of which articulate developmental mechanisms but have been limited by analytic constraints. ML’s capacity to detect complex, nonlinear interactions can help identify overlooked patterns and refine these theoretical models, contributing to improved developmental prediction and prevention strategies.

One goal of prevention science is to optimize health and behavioral outcomes by delivering the right intervention at the right time. Developmental cascade models add value by revealing the mechanisms and developmental timing through which early experiences shape later outcomes, enabling interventions to target the most influential pathways when change is most achievable (Otten et al., 2019; Wolchik et al., 2016). Incorporating ML into this framework extends these insights by supporting the creation of individualized risk profiles that inform tailored, population-specific interventions.

ML-enhanced developmental cascade models thus hold significant promise for prevention science. By linking data-driven discovery with theory-guided developmental modeling, this integrative approach can help researchers identify critical periods for intervention, allocate resources efficiently, and design prevention strategies that are both personalized and developmentally informed. Together, these advances position ML-integrated cascade models as a powerful tool for providing a scalable and theory-informed pathway for advancing precision prevention across the life course.

## Declarations

### Authors and Affiliations

Department of Public Health Sciences, University of Miami Miller School of Medicine, Miami, Florida, USA; Vanessa Morales, Francisco Cardozo, Daniel J. Feaster, Sara M. St. George, Raymond R. Balise.

## References

[CR1] Arlot, S., & Celisse, A. (2010). A survey of cross-validation procedures for model selection. *Statistics Surveys*, *4*(none). 10.1214/09-SS054

[CR2] Babyak, M. A. (2004). What you see may not be what you get: A brief, nontechnical introduction to overfitting in regression-type models. *Psychosomatic Medicine,**66*(3), 411–421. 10.1097/01.psy.0000127692.23278.a9 15184705

[CR3] Baribeau, D. A., Vigod, S., Brittain, H., Vaillancourt, T., Szatmari, P., & Pullenayegum, E. (2022). Application of transactional (cross-lagged panel) models in mental health research: An introduction and review of methodological considerations. *Journal of the Canadian Academy of Child and Adolescent Psychiatry = Journal De l’Academie Canadienne De Psychiatrie De l’Enfant Et De l’Adolescent,**31*(3), 124–134. 35919904 PMC9275371

[CR4] Beam, A. L., & Kohane, I. S. (2018). Big data and machine learning in health care. *JAMA,**319*(13), 1317. 10.1001/jama.2017.18391 29532063

[CR5] Biecek, P., & Burzykowski, T. (2021). *Explanatory model analysis: Explore, explain and examine predictive models* (1st ed.). Chapman and Hall/CRC. 10.1201/9780429027192

[CR6] Black, J. E., Kueper, J. K., & Williamson, T. S. (2023). An introduction to machine learning for classification and prediction. *Family Practice,**40*(1), 200–204. 10.1093/fampra/cmac104 36181463

[CR7] Breiman, L. (2001). Random forests. *Machine Learning,**45*(1), 5–32. 10.1023/A:1010933404324

[CR8] Byrne, B. M. (2016). *Structural equation modeling with AMOS: Basic concepts, applications, and programming, third edition* (0 ed.). Routledge. 10.4324/9781315757421

[CR9] Bzdok, D., Altman, N., & Krzywinski, M. (2018). Statistics versus machine learning. *Nature Methods,**15*(4), 233–234. 10.1038/nmeth.4642 30100822 PMC6082636

[CR10] Conduct Problems Prevention Research. (2011). The effects of the fast track preventive intervention on the development of conduct disorder across childhood. *Child Development,**82*(1), 331–345. 10.1111/j.1467-8624.2010.01558.x 21291445 PMC3682774

[CR11] Dishion, T. J., Véronneau, M.-H., & Myers, M. W. (2010). Cascading peer dynamics underlying the progression from problem behavior to violence in early to late adolescence. *Development and Psychopathology,**22*(3), 603–619. 10.1017/S0954579410000313 20576182

[CR12] Dodge, K. A., Greenberg, M. T., & Malone, P. S. (2008). Testing an idealized dynamic cascade model of the development of serious violence in adolescence. *Child Development,**79*(6), 1907–1927. 10.1111/j.1467-8624.2008.01233.x 19037957 PMC2597335

[CR13] Eiden, R. D., Lessard, J., Colder, C. R., Livingston, J., Casey, M., & Leonard, K. E. (2016). Developmental cascade model for adolescent substance use from infancy to late adolescence. *Developmental Psychology,**52*(10), 1619–1633. 10.1037/dev0000199 27584669 PMC5056785

[CR14] Ellis, R. J., Sander, R. M., & Limon, A. (2022). Twelve key challenges in medical machine learning and solutions. *Intelligence-Based Medicine,**6*, Article 100068. 10.1016/j.ibmed.2022.100068

[CR15] Frick, H., Chow, F., Kuhn, M., Mahoney, M., Silge, J., & Wickham, H. (2022). rsample [R package]. Posit / comprehensive R archive network. Retrieved November 20, 2025, from https://cran.r-project.org/package=rsample

[CR16] Geller, A., Jaeger, K., & Pace, G. (2018). Using the fragile families and child wellbeing study (FFCWS) in life course health development research. In N. Halfon, C. B. Forrest, R. M. Lerner, & E. M. Faustman (Eds.), Handbook of life course health development. Springer Retrieved November 20, 2025, from http://www.ncbi.nlm.nih.gov/books/NBK543725/31314304

[CR17] Gibbons, R. D., Hedeker, D., & DuToit, S. (2010). Advances in analysis of longitudinal data. *Annual Review of Clinical Psychology,**6*, 79–107. 10.1146/annurev.clinpsy.032408.153550 20192796 PMC2971698

[CR18] Goodfellow, I., Courville, A., & Bengio, Y. (2016). *Deep learning*. The MIT Press.

[CR19] Gottfredson, D. C., Cook, T. D., Gardner, F. E. M., Gorman-Smith, D., Howe, G. W., Sandler, I. N., & Zafft, K. M. (2015). Standards of evidence for efficacy, effectiveness, and scale-up research in prevention science: Next generation. *Prevention Science : The Official Journal of the Society for Prevention Research,**16*(7), 893–926. 10.1007/s11121-015-0555-x 25846268 PMC4579256

[CR20] Hamaker, E. L., Kuiper, R. M., & Grasman, R. P. P. P. (2015). A critique of the cross-lagged panel model. *Psychological Methods,**20*(1), 102–116. 10.1037/a0038889 25822208

[CR21] Harris, K. M., Halpern, C. T., Whitsel, E. A., Hussey, J. M., Killeya-Jones, L. A., Tabor, J., & Dean, S. C. (2019). Cohort profile: The national longitudinal study of adolescent to adult health (add health). *International Journal of Epidemiology,**48*(5), 1415k–1415kk. 10.1093/ije/dyz115 31257425 PMC6857761

[CR22] Harrison, K., Bost, K. K., McBride, B. A., Donovan, S. M., Grigsby-Toussaint, D. S., Kim, J., Liechty, J. M., Wiley, A., Teran-Garcia, M., & Jacobsohn, G. C. (2011). Toward a developmental conceptualization of contributors to overweight and obesity in childhood: The Six-Cs model: Developmental ecological model of child obesity. *Child Development Perspectives,**5*(1), 50–58. 10.1111/j.1750-8606.2010.00150.x

[CR23] Harrist, A. W., Topham, G. L., Hubbs‐Tait, L., Page, M. C., Kennedy, T. S., & Shriver, L. H. (2012). What developmental science can contribute to a transdisciplinary understanding of childhood obesity: An interpersonal and intrapersonal risk model. *Child Development Perspectives,**6*(4), 445–455. 10.1111/cdep.12004

[CR24] Hastie, T., Tibshirani, R., & Friedman, J. (2009). The elements of statistical learning. *Springer*. 10.1007/978-0-387-84858-7

[CR25] Hvitfeldt, E., & Silge, J. (2021). *Supervised machine learning for text analysis in R* (1st ed.). Chapman and Hall/CRC. 10.1201/9781003093459

[CR26] James, G., Witten, D., Hastie, T., & Tibshirani, R. (2021). An introduction to statistical learning: With applications in R. *Springer, US.* 10.1007/978-1-0716-1418-1

[CR27] James, G., Witten, D., Hastie, T., Tibshirani, R., & Taylor, J. (2023). An introduction to statistical learning: With applications in Python. *Springer International Publishing*. 10.1007/978-3-031-38747-0

[CR28] Keras: Multi-backend Keras (Version 3.12.0) [Python; MacOS, Unix]. (n.d.). Retrieved November 20, 2025, from https://pypi.org/project/keras/

[CR29] Klopack, E. T., & Wickrama, K. (2020). Modeling latent change score analysis and extensions in Mplus: A practical guide for researchers. *Structural Equation Modeling: A Multidisciplinary Journal,**27*(1), 97–110. 10.1080/10705511.2018.1562929 33013155 PMC7531193

[CR30] Kuhn, M. & Silge, J. (n.d.). Tidy modeling with R. Retrieved March 1, 2025, from https://www.tmwr.org/

[CR31] Kuhn, M., & Frick, H. (2022). multilevelmod: Model wrappers for multi-level models (Version 1.0.0) [R package]. Retrieved November 20, 2025, from https://cran.r-project.org/package=multilevelmod

[CR32] Kwok, O.-M., Chien, H. Y., Zhang, Q., Chang, C.-N., Elliott, T. R., & Bell, A.-S. (2024). Parallel processing modeling in longitudinal designs: An example predicting trajectories of distress and life satisfaction. *Rehabilitation Psychology,**69*(4), 301–314. 10.1037/rep0000545 38483536

[CR33] Lang, M., Binder, M., Richter, J., Schratz, P., Pfisterer, F., Coors, S., Au, Q., Casalicchio, G., Kotthoff, L., & Bischl, B. (2019). mlr3: A modern object-oriented machine learning framework in R. *Journal of Open Source Software,**4*(44), Article 1903. 10.21105/joss.01903

[CR34] Lee, T. K., Wickrama, K. K. A. S., & O’Neal, C. W. (2018). Application of latent growth curve analysis with categorical responses in social behavioral research. *Structural Equation Modeling: A Multidisciplinary Journal,**25*(2), 294–306. 10.1080/10705511.2017.1375858 31097902 PMC6516863

[CR35] LeWinn, K. Z., Caretta, E., Davis, A., Anderson, A. L., & Oken, E. (2022). SPR perspectives: Environmental influences on Child Health Outcomes (ECHO) Program: Overcoming challenges to generate engaged, multidisciplinary science. *Pediatric Research,**92*(5), 1262–1269. 10.1038/s41390-021-01598-0 34131290 PMC8204620

[CR36] Lier, P., & Koot, H. M. (2010). Developmental cascades of peer relations and symptoms of externalizing and internalizing problems from kindergarten to fourth-grade elementary school. *Development and Psychopathology,**22*(3), 569–582. 10.1017/S0954579410000283 20576179

[CR37] Liu, J., Pan, Y., Nelson, M. C., Gooden, L. K., Metsch, L. R., Rodriguez, A. E., Tross, S., Del Rio, C., Mandler, R. N., & Feaster, D. J. (2023). Strategies of managing repeated measures: Using synthetic random forest to predict HIV viral suppression status among hospitalized persons with HIV. *AIDS and Behavior,**27*(9), 2915–2931. 10.1007/s10461-023-04015-1 36739589 PMC10403627

[CR38] Lundberg, S., & Lee, S.-I. (2017). A unified approach to interpreting model predictions (version 2). *arXiv*. 10.48550/ARXIV.1705.07874

[CR39] Masiero, M., Spada, G. E., Sanchini, V., Munzone, E., Pietrobon, R., Teixeira, L., Valencia, M., Machiavelli, A., Fragale, E., Pezzolato, M., & Pravettoni, G. (2023). A machine learning model to predict patients’ adherence behavior and a decision support system for patients with metastatic breast cancer: Protocol for a randomized controlled trial. *JMIR Research Protocols,**12*, Article e48852. 10.2196/48852 38096002 PMC10755656

[CR40] Masten, A. S., & Cicchetti, D. (2010). Developmental cascades. *Development and Psychopathology,**22*(3), 491–495. 10.1017/S0954579410000222 20576173

[CR41] Molenaar, P. C. M. (2004). A manifesto on psychology as idiographic science: Bringing the person back into scientific psychology, this time forever. *Measurement: Interdisciplinary Research & Perspective,**2*(4), 201–218. 10.1207/s15366359mea0204_1

[CR42] Molnar, C. (2022). Interpretable machine learning: A guide for making black box models explainable (2nd ed.). Retrieved November 20, 2025, from https://christophm.github.io/interpretable-ml-book/

[CR43] Opitz, J. (2024). A closer look at classification evaluation metrics and a critical reflection of common evaluation practice. *Transactions of the Association for Computational Linguistics,**12*, 820–836. 10.1162/tacl_a_00675

[CR44] Osborne, J. W. (2015). Best practices in logistic regression. *SAGE Publications, Ltd.* 10.4135/9781483399041

[CR45] Otten, R., Mun, C. J., Shaw, D. S., Wilson, M. N., & Dishion, T. J. (2019). A developmental cascade model for early adolescent‐onset substance use: The role of early childhood stress. *Addiction,**114*(2), 326–334. 10.1111/add.14452 30280443 PMC6519208

[CR46] Oudekerk, B. A., Allen, J. P., Hessel, E. T., & Molloy, L. E. (2015). The cascading development of autonomy and relatedness from adolescence to adulthood. *Child Development,**86*(2), 472–485. 10.1111/cdev.12313 25345623 PMC4376599

[CR47] Pedregosa, F., Varoquaux, G., Gramfort, A., Michel, V., Thirion, B., Grisel, O., Blondel, M., Müller, A., Nothman, J., Louppe, G., Prettenhofer, P., Weiss, R., Dubourg, V., Vanderplas, J., Passos, A., Cournapeau, D., Brucher, M., Perrot, M., & Duchesnay, É. (2012). *Scikit-learn: Machine learning in Python*. 10.48550/ARXIV.1201.0490

[CR48] Pudjihartono, N., Fadason, T., Kempa-Liehr, A. W., & O’Sullivan, J. M. (2022). A review of feature selection methods for machine learning-based disease risk prediction. *Frontiers in Bioinformatics,**2*, Article 927312. 10.3389/fbinf.2022.927312 36304293 PMC9580915

[CR49] Quinlan, J. R. (1986). Induction of decision trees. *Machine Learning,**1*(1), 81–106. 10.1007/BF00116251

[CR50] Rasmussen, C. E., & Williams, C. K. I. (2005). Gaussian processes for machine learning. *The MIT Press*. 10.7551/mitpress/3206.001.0001

[CR51] Ribeiro, M. T., Singh, S., & Guestrin, C. (2016). Why should I trust you?”: Explaining the predictions of any classifier (Version 3). *arXiv*. 10.48550/ARXIV.1602.04938

[CR52] Rickert, J. (2023). Multistate models for medical applications. R views. Retrieved November 20, 2025, from https://rviews.rstudio.com/2023/04/19/multistate-models-for-medical-applications/

[CR53] Saito, T., & Rehmsmeier, M. (2015). The precision-recall plot is more informative than the ROC plot when evaluating binary classifiers on imbalanced datasets. *PLoS ONE,**10*(3), Article e0118432. 10.1371/journal.pone.0118432 25738806 PMC4349800

[CR54] Saragosa-Harris, N. M., Chaku, N., MacSweeney, N., Guazzelli Williamson, V., Scheuplein, M., Feola, B., Cardenas-Iniguez, C., Demir-Lira, E., McNeilly, E. A., Huffman, L. G., Whitmore, L., Michalska, K. J., Damme, K. S., Rakesh, D., & Mills, K. L. (2022). A practical guide for researchers and reviewers using the ABCD Study and other large longitudinal datasets. *Developmental Cognitive Neuroscience,**55*, Article 101115. 10.1016/j.dcn.2022.101115 35636343 PMC9156875

[CR55] Schober, P., & Vetter, T. R. (2021). Linear regression in medical research. *Anesthesia and AnalgesIa,**132*(1), 108–109. 10.1213/ANE.0000000000005206 33315608 PMC7717471

[CR56] Scikit-learn: A set of Python modules for machine learning and data mining (Version 1.7.2) [C, Python; MacOS, Windows, POSIX, Unix]. (n.d.). Retrieved November 20, 2025, from https://scikit-learn.org/

[CR57] Smith, J. D., Egan, K. N., Montaño, Z., Dawson-McClure, S., Jake-Schoffman, D. E., Larson, M., & St George, S. M. (2018). A developmental cascade perspective of paediatric obesity: A conceptual model and scoping review. *Health Psychology Review,**12*(3), 271–293. 10.1080/17437199.2018.1457450 29583070 PMC6324843

[CR58] Sokolova, M., & Lapalme, G. (2009). A systematic analysis of performance measures for classification tasks. *Information Processing & Management,**45*(4), 427–437. 10.1016/j.ipm.2009.03.002

[CR59] TensorFlow: TensorFlow is an open-source machine learning framework for everyone (Version 2.20.0) [Python]. (n.d.). Retrieved November 22, 2025, from https://www.tensorflow.org/

[CR60] The Top Programming Languages 2025—IEEE Spectrum. (n.d.). IEEE Spectrum. Retrieved November 20, 2025, from https://spectrum.ieee.org/top-programming-languages-2025

[CR61] Tibshirani, R. (2011). Regression shrinkage and selection via the Lasso: A retrospective. *Journal of the Royal Statistical Society. Series B, Statistical Methodology,**73*(3), 273–282. 10.1111/j.1467-9868.2011.00771.x

[CR62] tidymodels. (n.d.). themis: Extra recipes steps for dealing with unbalanced data. GitHub. Retrieved November 20, 2025, from https://github.com/tidymodels/themis

[CR63] TIOBE Index. (n.d.). *TIOBE*. Retrieved November 20, 2025, from https://www.tiobe.com/tiobe-index/

[CR64] Van Den Goorbergh, R., Van Smeden, M., Timmerman, D., & Van Calster, B. (2022). The harm of class imbalance corrections for risk prediction models: Illustration and simulation using logistic regression. *Journal of the American Medical Informatics Association,**29*(9), 1525–1534. 10.1093/jamia/ocac093 35686364 PMC9382395

[CR65] Vaughan, D., Couch, S., & Frick, H. (n.d.). workflows: Modeling workflows (R package version 1.3.0). Posit Software, PBC. https://workflows.tidymodels.org

[CR66] Vovk, V., Gammerman, A., & Shafer, G. (Eds.). (2005). *Algorithmic learning in a random world*. Springer Science+Business Media, Inc. 10.1007/b106715

[CR67] Wiedermann, W., Bonifay, W., & Huang, F. L. (2023). Advanced categorical data analysis in prevention science. *Prevention Science,**24*(3), 393–397. 10.1007/s11121-022-01485-y 36633766

[CR68] Wolchik, S. A., Tein, J.-Y., Sandler, I. N., & Kim, H.-J. (2016). Developmental cascade models of a parenting-focused program for divorced families on mental health problems and substance use in emerging adulthood. *Development and Psychopathology,**28*(3), 869–888. 10.1017/S0954579416000365 27427811 PMC5444389

[CR69] Zakeri, M., Sansgiry, S. S., & Abughosh, S. M. (2022). Application of machine learning in predicting medication adherence of patients with cardiovascular diseases: A systematic review of the literature. *Journal of Medical Artificial Intelligence,**5*, 5–5. 10.21037/jmai-21-26

